# *Tomato leaf curl Kerala virus *(ToLCKeV) AC3 protein forms a higher order oligomer and enhances ATPase activity of replication initiator protein (Rep/AC1)

**DOI:** 10.1186/1743-422X-7-128

**Published:** 2010-06-14

**Authors:** Kalyan K Pasumarthy, Nirupam R Choudhury, Sunil K Mukherjee

**Affiliations:** 1 International Centre for Genetic Engineering and Biotechnology, Aruna Asaf Ali Marg, New Delhi -110067, India

## Abstract

**Background:**

Geminiviruses are emerging plant viruses that infect a wide variety of vegetable crops, ornamental plants and cereal crops. They undergo recombination during co-infections by different species of geminiviruses and give rise to more virulent species. Antiviral strategies targeting a broad range of viruses necessitate a detailed understanding of the basic biology of the viruses. ToLCKeV, a virus prevalent in the tomato crop of Kerala state of India and a member of genus Begomovirus has been used as a model system in this study.

**Results:**

AC3 is a geminiviral protein conserved across all the begomoviral species and is postulated to enhance viral DNA replication. In this work we have successfully expressed and purified the AC3 fusion proteins from *E. coli*. We demonstrated the higher order oligomerization of AC3 using sucrose gradient ultra-centrifugation and gel-filtration experiments. In addition we also established that ToLCKeV AC3 protein interacted with cognate AC1 protein and enhanced the AC1-mediated ATPase activity in vitro.

**Conclusions:**

Highly hydrophobic viral protein AC3 can be purified as a fusion protein with either MBP or GST. The purification method of AC3 protein improves scope for the biochemical characterization of the viral protein. The enhancement of AC1-mediated ATPase activity might lead to increased viral DNA replication.

## Background

Tomato leaf curl disease (ToLCD) is a cause of concern for the tomato plant. This disease is mostly caused by leaf curl viruses of *Geminiviridae *family that include more than 50 species of Tomato leaf curl viruses of genus Begomovirus. Threat to the tomato crop is further aggravated by the high level of recombination observed in the geminiviruses during mixed infection resulting in the emergence of new and virulent viral species. Various approaches have been adopted to control the geminiviruses through traditional breeding and transgenic approaches. Noted among them are transgenic approaches based on viral intergenic sequences [1], mutant viral proteins [2-7], antisense RNAs [8,9] and peptide aptamers [10,11]. But most of them have been either less efficient at the field level or are limited to narrow range of virus species. Thus, there is a need for a better and consistent approach to generate resistant plants against a broad range of virus species.

Understanding the basic biology, such as replication of the geminiviruses expands the scope of the development of antiviral strategies to target the viral infection. Geminiviruses possess closed circular ssDNA (~ 2.7 kb) and the virion particles are transferred from one plant to another by the plant vectors like leaf hopper and white fly. Geminiviruses replicate via rolling circle replication. Various studies have shown that the viral proteins, AC1/C1 and AC3/C3 are required for the viral replication [12-18]. While AC1 was well characterized for its role in initiation, elongation and termination of replication [19-21], little information is available regarding the characteristics of AC3/C3 protein of geminiviruses. AC3/C3 was shown to enhance DNA replication in protoplast assays and leaf disc assays [14,22]. However, the mechanism of replication enhancement by AC3, its structure and biochemical properties are under explored due to the difficulty associated with the purification of soluble AC3 protein [23]. To better understand the biochemical characteristics of the AC3 protein, we have developed a robust prokaryotic expression system for Tomato leaf curl Kerala virus-[India:Kerala II:2005] (ToLCKeV; DQ852623) AC3 protein in *E. coli *and studied its oligomeric properties in solution. We have also examined the interaction of ToLCKeV AC3 with the cognate AC1 protein and the impact of this interaction on the ATPase activity of AC1.

## Results and Discussion

### Recombinant protein expression and purification of ToLCKeV AC3 and AC1 proteins

**Figure 1:**
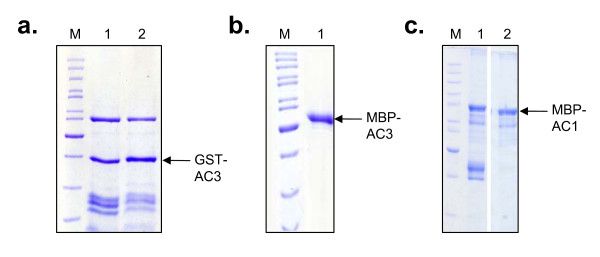
**Expression and purification of recombinant AC3 and AC1 proteins**. (a) Purified GST-AC3 fusion protein. (b) Purified MBP-AC3 fusion protein. (c) Purified MBP-AC1 fusion protein. All the proteins were run on separate SDS-PAGE gels and stained with Coomassie blue. 'M' denotes the marker lane and protein from different batches are denoted by numbers.

ToLCKeV AC3 is a 134 aa protein and is highly hydrophobic (84/134 aa) like its homologs such as Tomato yellow leaf curl virus (TYLCV) C3 and Tomato golden mosaic virus (TGMV) AC3 proteins [22]. High hydrophobicity of the AC3 protein makes it unstable in the solution and the protein forms inclusion bodies when expressed with His tag [[23], unpublished data from our lab]. Instability might also be due to the poor recruitment of molecular chaperones at the site of protein synthesis in prokaryotic cells. However, AC3 could be expressed from insect cells as a GST fusion protein [24]. Since the use of insect cell line is expensive, we expressed AC3 protein as a fusion with glutathione S-transferase (GST) and maltose binding protein (MBP) in *E. coli *(Fig 1a,b). MBP is known to facilitate the proper folding of the fusion protein by acting as an intra-molecular chaperone [25] and the reason for choosing GST is the ease in performing in vitro protein-protein interaction studies. GST-AC3 and MBP-AC3 protein possessed a molecular mass of 40 kDa and 55 kDa which differed marginally from the predicted value (41.8 kDa and 58 kDa respectively) of the fusion ORF. A similar kind of deviation in the mobility on SDS-PAGE was observed in case of His-AC3 protein from TGMV [23] and thus, it seems to be inherent to all the AC3 fusion proteins. Our attempts to release the AC3 protein by cleaving it from the MBP fusion resulted in precipitation of AC3 protein (data not shown). So, we proceeded with the MBP-AC3 in our studies.

Similarly, ToLCKeV AC1 was also expressed with His tag and GST fusion. However, ToLCKeV AC1 could not be expressed as a soluble protein in either case (data not shown). A similar case was observed in case of MYMIV-sp [MP] Rep protein [26]. So, we cloned the Rep protein as an MBP fusion and purified it as the soluble protein (Fig 1c).

### ToLCKeV AC3 protein forms a higher order oligomer

**Figure 2:**
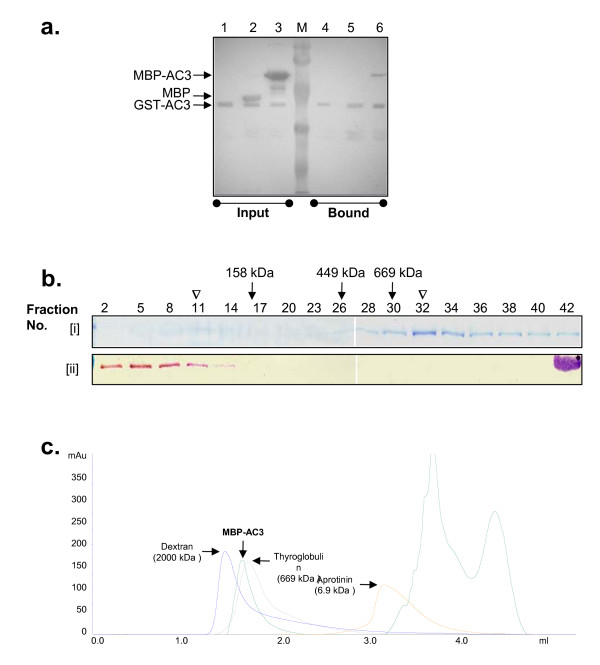
**ToLCKeV AC3 forms a higher order oligomer**. (a) Western blotting of GST pull-down assay using poly-clonal MBP-AC3 antibodies. Fractions corresponding to 'input' represent the protein composition of the total reaction mix for protein-protein interactions. Fractions corresponding to 'bound' represent the proteins that are interacting with GST-AC3 bound to glutathione sepharose. Presence of MBP-AC3 in the bound fraction indicates the formation of oligomer. (b) [i] Protein distribution pattern for the MBP-AC3 after sucrose gradient ultracentrifugation was visualised by Coomassie blue staining. MBP-AC3 forms a faint peak at 11 ^th ^fraction and a prominent peak at 32 ^nd ^fraction as indicated by '**V**'. Arrows indicate the peak formation of molecular weight standard proteins: Aldolase (158 kDa) at 17 ^th ^fraction, Ferritin (449 kDa) at 26 ^th ^fraction and Thyroglobulin (669 kDa) at 30 ^th ^fraction. [ii] MBP (43 kDa) does not form an oligomer and peaks in the 5 ^th ^fraction. (c) Gel filtration with Superdex-200 5/150 column shows the elution of various proteins. MBP-AC3 elutes between the Dextran (2000 kDa) and Thyroglobulin (669 kDa).

Replication is a complex process that involves interaction with various proteins. Many a times self-oligomerization of a protein generates multiple sites to interact or increase the area of interaction, thereby strengthening the interaction. Since, AC3 was known to play role in replication and new world viral protein TGMV AC3 is known to oligomerize [22,24], we examined if the old world viral protein ToLCKeV AC3 could also be oligomeric in nature. We performed an in vitro GST pull-down reaction with the GST-AC3 and MBP-AC3 proteins in the solution (Fig 2a). The fact that MBP and GST does not play any role in these interaction studies was confirmed through the control reactions performed along with the test reaction. In these in vitro reactions, MBP alone was not observed in the bound fractions containing GST-AC3 (Fig 2a, lanes 2 and 5). However, MBP-AC3 was observed in the bound fraction containing the GST-AC3 (Fig 2a, lanes 3 and 6), which is possible only through the inter-molecular interaction between AC3 molecules. Though this experiment corroborated with the observations that the ToLCKeV AC3 oligomerized like the new world TGMV AC3 [24], the oligomeric status could not be inferred from this experiment alone.

Preliminary experiments with TGMV AC3 indicated that it forms a higher order oligomer of more than 100 kDa which has not been deciphered further [22]. So, to find the exact oligomeric status of AC3 protein, we opted for the sucrose gradient ultracentrifugation method and gel-filtration with the purified ToLCKeV AC3 protein. The reaction mixture of MBP-AC3 was loaded onto the step gradient of sucrose which was centrifuged as described in the 'Methods' section along with control protein MBP and molecular weight markers in separate tubes. In this experiment, a comparison with the control molecular weight markers together with the gel-filtration data indicated that MBP-AC3 most probably existed as an oligomer with a molecular mass higher than 669 kDa which corresponds to a higher order oligomer of 12-14 mer (Fig 2b,c). Since, the control protein MBP does not show the higher order oligomeric formation [Fig 2b (ii)], the higher molecular weight observed in case of fusion protein MBP-AC3 can be attributed to the oligomerization of the recombinant AC3 protein alone.

### AC3 interacts with AC1 in vitro and enhances the ATPase activity of AC1

Previous studies in tobacco protoplasts with TGMV AC3 indicated that it either facilitates or stabilizes the AC1-DNA interaction [27]. Other studies have also indicated that AC1 interacts with AC3 co-expressed in yeast or insect cells [22,24]. However, the impact of this interaction was not investigated in its biochemical terms. So, we asked if ToLCKeV AC3 interacts with AC1 in vitro, and what could be its effect on the biochemical activity of AC1, particularly the ATPase activity of AC1.

**Figure 3:**
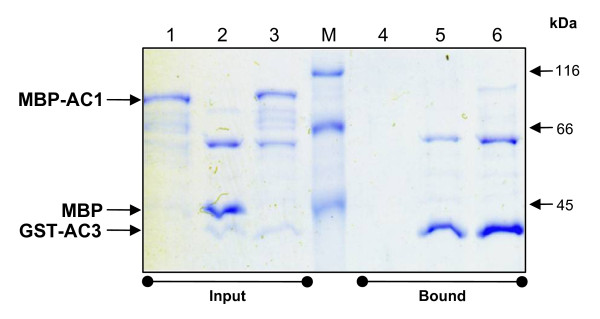
**GST pull-down assay for in vitro interaction of ToLCKeV GST-AC3 and MBP-AC1**. Coomassie blue stained SDS-PAGE showing in vitro interaction between GST-AC3 and MBP-AC1. Fractions corresponding to 'input' (lanes 1-3) represent the total protein composition in each reaction mix. GST-AC3 bound proteins were thoroughly washed to remove the non-specifically interacting proteins. 'Bound' fractions (lanes 4-6) represent the proteins that were interacting with GST-AC3. Presence of AC1 in the bound fraction (lane 6) indicates its interaction with AC3.

We have performed an in vitro GST pull-down assay with MBP-AC1 and GST-AC3 along with the control reactions (Fig 3). We observed that MBP-AC1 is present only in the bound fraction in the presence of GST-AC3 (Fig 3, lanes 3 and 6) whereas MBP-AC1 fusion protein alone (Fig 3, lanes 1 and 4) or MBP alone is unable to bind to the glutathione resin (Fig 3, lanes 2 and 5). These control reactions indicated that the interaction observed with GST-AC3 and MBP-AC1 could be possible only if AC1 and AC3 interacted with each other. This interaction study done with the recombinant proteins purified from *E. coli *corroborated with the earlier experiments done with the TGMV AC3 and TGMV AC1 proteins co-expressed in insect cell lines [24] and the yeast two hybrid experiments carried out with TYLCV C3 and TYLCV C1 [22].

AC1 protein is multi-functional protein with DNA binding activity [27-32], site-specific DNA nicking activity [33,34], ATPase activity [19,20,35], helicase activity [36,37] and also modulates the gene expression from the complementary strand of the viral DNA [38-42]. The in vitro interaction with the purified AC3 and AC1 fusion proteins prompted us to question the after effects of this interaction on the biochemical activity of AC1. ATPase activity is an important property of AC1 and affects the site-specific DNA nicking and ligation activity [43] and is also implicated in the helicase activity [36,37]. Hence, we investigated if AC3 interaction with AC1 had any effect on AC1-mediated ATPase activity.

**Figure 4:**
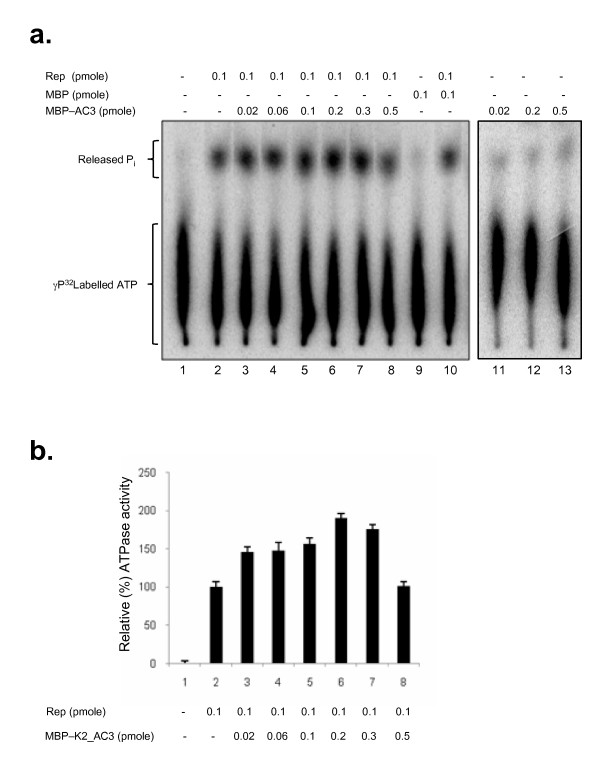
**AC3 modulates the ATPase activity of Rep**. (a) Autoradiograph showing the ATPase activity of Rep in the absence and presence of AC3. AC3 increases the ATPase activity of Rep at low concentration (0.02-0.2 pM) by 50-80%. Composition of the proteins in the reaction mix is shown at the top of each lane in the autoradigraph. ATPase reaction was carried with a uniform concentration of Rep protein and varying concentrations of MBP-AC3 as denoted in the figure. MBP was used as a negative control. (b) Graphical representation of ATPase activity of Rep in the presence of MBP-AC3. ATPase activity in the reaction mix containing the Rep protein alone was arbitrarily assigned a value of 100% and activity in other lanes was calculated accordingly. Graph was plotted for the lanes 1-8 that correspond to the lanes of autoradiograph.

A series of ATPase reactions were performed to assess the influence of AC3 on AC1-ATPase activity (Fig 4a). Comparison of control reactions with MBP protein alone (Fig 4a, lane 9), MBP with AC1 (Fig 4a, lane 10), MBP-AC3 alone (Fig 4a, lanes 11, 12, 13) did not show any significant ATPase activity in the reaction. However, in the presence of MBP-AC3, AC1 protein revealed a significant increase in the ATPase activity which can be attributed to AC3 interaction with AC1 (Fig 4a, lanes 3-6). ATPase activity enhanced to about 50%-80% initially and reduced upon further increase in the concentration of AC3 protein in the reaction mix resulting in a bell shaped curve (Fig 4b). Modulation of ATPase activity is significant in the context of the multi-functional role of AC1. ATPase activity is necessary for the helicase activity of Rep which was also proposed to be a likely replicative helicase [36,37]. In this context the modulation of ATPase activity by AC3 assumes significance as it might influence the AC1's helicase activity and gives a direction on the way AC3 enhances the replication. Further studies of the role of AC3 in modulating the role of helicase activity are under investigation.

## Methods

### Cloning, expression and purification of recombinant MBP and GST fusion proteins

AC3 and AC1 ORFs were amplified from Tomato leaf curl Kerala virus-[India:Kerala II:2005] (NCBI Accession No. DQ852623) using degenerate oligos designed from the CLUSTALW multiple alignment of AC3 and AC1 ORFs from various geminiviral genomes isolated in our lab (DQ629101, DQ629102, DQ629103, DQ887537, AJ314739, AF126406). Following oligos were used in the current experiments:

All_AC3_Fwd: 5'- CATGAGCTCGGATCCATGGATTCACGCACAGGG -3'

All_AC3_Rev: 5'- CCATCTAGACTCGAGTGGCRTGTACTCAYGCCTCTAAYCC -3'

ToLCV_AC1_Fwd: 5'- CATGGATCCATGGCHVCYCCMAAWCG -3'

ToLCV_AC1_Rev: 5'- TGACTCGAGTCAACYCGWCGACGHCTGG -3'

Amplified ORFs were purified from the PCR mix and cloned into pGEMT-Easy cloning vector. The cloned vectors were then digested with BamH I and Sal I restriction enzymes. The digested ORFs were purified and sub-cloned into BamH I and Sal I digested pMal-c2X and pGEX-4T-1. Expression vectors containing AC3 and AC1 ORFs were then transformed into *E. coli*. The bacterial cells containing the expression vectors were induced over-night at 18°C with 0.01 mM IPTG. The induced cells were harvested and sonicated as per standard methods. The MBP fusion proteins and GST fusion protein were purified by affinity chromatography with amylose resin and glutathione sepharose respectively as per the manufacturer's protocol (New England Biolabs and GE Healthcare respectively). Purified proteins were dialysed in buffer containing 50 mM Tris, 100 mM NaCl and 40% glycerol and the proteins were stored in aliquots at -20°C.

### GST pull-down assay

Purified GST fusion protein was incubated with varied amounts of MBP fusion protein in binding buffer [25 mM Tris (pH 8.0), 75 mM NaCl, 2.5 mM EDTA, 5 mM MgCl_2_, 2.5 mM DTT, 1% NP-40] at 37°C for 30 min. Glutathione sepharose 4B resin was equilibrated with binding buffer and 10 μl of resin was added to the incubated protein mixture and kept on nutator for 30 min. Unbound protein fraction was separated from the resin by centrifugation at 3,000×g for 3 min. Resin bound to the protein was washed with increasing concentrations of NaCl (100 mM to 400 mM) in binding buffer. Equal amount of 2× sample loading buffer [100 mM Tris-HCl (pH 6.8), 200 mM DTT, 4% SDS, 0.2% Bromophenol blue] was then added to the resin, boiled for 5 min, centrifuged briefly and the supernatant was analyzed by SDS-PAGE. The protein bands were visualized by western blotting or Coomassie blue staining as per standard procedures.

### Sucrose Gradient

We followed the protocol that was used for the analysis of oligomerization of Rep [36]. About 250 mg of each of the purified proteins was layered directly on a 10.5 ml of 10-40% (w/v) sucrose step gradient in a buffer containing 25 mM Tris (pH 8.0), 250 mM NaCl, 2 mM Sodium bisulphite and 0.05% Triton X-100. Gradients were centrifuged in a Beckman SW41Ti rotor at 35,000 rpm for 20 h at 4°C. Fractions (250 μl) were collected and subjected to 10% SDS-PAGE. The protein bands were visualized by silver staining. Protein molecular mass markers viz., Aldolase (158 kDa), Ferritin (449 kDa), and Thyroglobulin (669 kDa) were run in parallel gradients. Each fraction of 250 μl represented a sedimentation distance of 2.12 mM as an 11 ml solution filled up an axial length of 89 mM in the centrifuge tube. The sedimentation distance (y in mm) corresponding to a fraction 'f' was represented by the equation y = 67+2.12×'f', where 67 is the distance from the axis of rotation to the top of the centrifuge tube. Regression analysis using the Microsoft Excel application program yielded the equation: y = 35.490+29.754×Log (x); R^2 ^= 0.997, where y represents the sedimentation distance (in mm) and x represents the molecular mass (in kDa). The sedimentation distance for MBP-AC3 was fitted into the standard curve and their native molecular mass was estimated.

### Gel Filtration

Oligomerization status of AC3 was analyzed with gel filtration using Superdex 200 5/150 column in Acta Prime (GE Healthcare) having a bed volume of 3 ml and a void volume of 1.374 ml. Protein sample (100 μl) was injected and the flow rate of the column was maintained at 200 μl per minute all through the process. Dextran (2000 kDa), Thyroglobulin (669 kDa), Ferritin (449 kDa), Aldolase (158 kDa) and Aprotinin (9 kDa) were used as molecular weight standards under the same conditions.

### ATPase Assay

The ATPase reaction was carried out by incubating the radiolabeled ATP [10 μCi of (γ- ^32^P) ATP (6000 Ci/mmol) was diluted 50 fold with 5 mM ATP] with Rep and/or MBP-AC3 in buffered solution [20 mM Tris-Cl (pH 8.0), 1 mM MgCl_2_, 100 mM KCl, 8 mM DTT, and 80 ng/μl of BSA] for 30 min at 37°C. After the reaction, 1 μl of the reaction mix was spotted on PEI-TLC plate. Plate was air-dried and chromatographed with a running solvent (0.5 M LiCl and 1 M HCOOH). Following completion of chromatography, TLC paper was dried and autoradiographed. The relative intensities of the released Pi were estimated by densitometric scanning using Typhoon 9210 scanner and analyzed by ImageQuant TL software (GE Healthcare, UK).

## Conclusions

In this study, we have successfully purified the highly hydrophobic geminiviral AC3 protein from the laboratory strain of *E. coli *BL21(DE3). The purified protein was successfully utilized in the biochemical characterization studies. We observed that AC3 forms a higher order oligomer like the AC1 protein from other geminiviruses. AC3 interacted with AC1 but not with mole to mole ratio, indicating self-interaction might predominate over hetero-interaction. The observation that AC3 enhances the ATPase activity of AC1 gives light on the way AC3 enhances viral DNA replication. At higher concentration, AC3 failed to upregulate AC1-mediated ATPase activity indicating that AC1 might not gain proper access to self-oligomeric AC3.

## Competing interests

The authors declare that they have no competing interests.

## Authors' contributions

KKP had done all the experiments and drafted the manuscript. NRC helped in the sucrose gradient ultracentrifugation experiment. KKP, NRC and SKM together designed the experiments. NRC and SKM had proof-read and finalized the manuscript. All authors read and approved the final manuscript.
